# Lymph Node Findings Following Axillary Lymph Node Dissection in Cases of a Positive Sentinel Lymph Node in Breast Cancer

**DOI:** 10.7759/cureus.109447

**Published:** 2026-05-22

**Authors:** Christos Kalyvopoulos, George Simatos, Evangelos P Solakis, George Intas, George Kinoglou, Aristofania Simatou, Evangelia Skafida, Dimitris Tryfonopoulos, George Rigas

**Affiliations:** 1 First Department of Breast Surgery, Anti-Cancer Hospital of “Saint Savvas”, Athens, GRC; 2 Department of General Surgery, 401 General Military Hospital of Athens, Athens, GRC; 3 Department of Nursing, General Hospital of Nikaia, Athens, GRC; 4 Second Department of Oncology, Anti-Cancer Hospital of “Saint Savvas”, Athens, GRC; 5 Department of Pathological Anatomy, Anti-Cancer Hospital of “Saint Savvas”, Athens, GRC

**Keywords:** axillary lymph node dissection, breast cancer, breast cancer research, de-escalation, sentinel lymph node, sentinel lymph node biopsy, surgical oncology

## Abstract

Background

Axillary management in breast cancer has undergone significant evolution over recent decades. Sentinel lymph node biopsy (SLNB) has gradually replaced conventional axillary lymph node dissection (ALND) as the preferred staging procedure in clinically node-negative patients, primarily due to its lower morbidity. The role of completion ALND in patients with a positive sentinel lymph node (SLN) remains an area of active investigation, particularly in light of trials supporting less aggressive axillary management. This study aimed to describe the frequency and extent of additional non-sentinel axillary lymph node involvement following completion ALND in patients with a positive SLN, and to explore clinicopathological associations with additional nodal burden. The study was designed as a descriptive and exploratory retrospective analysis.

Methodology

A retrospective, single-center study was conducted among 102 female patients with histologically confirmed breast cancer who underwent surgery between 2018 and 2019 and were found to have a positive SLN. Data were retrieved from the institutional oncology registry. Patient and tumor characteristics recorded included age, type of breast surgery (breast-conserving surgery (BCS) or mastectomy), TNM staging, histological subtype, tumor grade, hormone receptor (HR) status (estrogen receptor and progesterone receptor), human epidermal growth factor receptor 2 (HER2) status, and Ki-67 proliferation index. Notably, no patient in this cohort received neoadjuvant systemic therapy before surgery. Lymphovascular invasion data were not available in the registry and represent a limitation of this study. Statistical analysis included descriptive and inferential methods using SPSS version 22.0, with p-values <0.05 considered significant.

Results

The mean patient age was 57.2 ± 13.2 years (range = 28-85). The majority of patients underwent BCS (79.4%, n = 81) rather than mastectomy (20.6%, n = 21). Tumor staging revealed T1 disease in 32.4% (n = 33), T2 in 46.1% (n = 47), and T3 in 5.9% (n = 6); staging data were unavailable for 15.7% of patients. The predominant molecular subtype was luminal (HR+/HER2-) in 75.5% (n = 77), followed by HER2+ (HR+) in 8.8% (n = 9), triple negative in 8.8% (n = 9), and HER2+ (HR-) in 6.9% (n = 7). Tumor grade was 2 in 52.9% (n = 53), grade 3 in 42.2% (n = 43), and grade 1 in 2.0% (n = 2). On average, 3.01 ± 2.4 SLNs were removed (mean positive = 1.7 ± 1.3), and 11.4 ± 5.2 axillary lymph nodes were removed during completion ALND (mean positive = 2.6 ± 3.7). Additional non-sentinel axillary lymph node metastases were identified in 58.8% (n = 60) of patients, while 41.2% (n = 42) had no further nodal disease. Patients with invasive carcinoma demonstrated significantly higher nodal burden (p < 0.05). No statistically significant associations were identified between ALND positivity and age, surgery type, tumor stage, grade, or molecular subtype in this cohort, likely reflecting limited statistical power.

Conclusions

In this retrospective, single-center study, 58.8% of patients with a positive SLN had additional axillary metastases following completion ALND, underscoring that residual nodal disease remains common and clinically significant. Conversely, 41.2% had no further axillary involvement, consistent with literature supporting selective de-escalation of axillary surgery in carefully selected patients.

## Introduction

Breast cancer is the most commonly diagnosed malignancy among women worldwide and a leading cause of cancer-related mortality [1,2]. Axillary lymph node status is among the most important prognostic factors, informing decisions regarding systemic therapy, radiotherapy, and surgical extent [1].

Over recent decades, sentinel lymph node biopsy (SLNB) has largely replaced axillary lymph node dissection (ALND) as the staging procedure of choice in clinically node-negative early breast cancer. SLNB provides reliable nodal staging with substantially lower morbidity, including reduced rates of lymphedema, shoulder dysfunction, and chronic pain [3,4].

The management of patients with a positive sentinel lymph node (SLN) has evolved considerably. Landmark trials including ACOSOG Z0011 and IBCSG 23-01 demonstrated that omission of completion ALND (cALND) in selected patients, those with one or two positive SLNs undergoing breast-conserving surgery (BCS) with adjuvant whole-breast radiotherapy, did not compromise locoregional control or overall survival [5-8]. The AMAROS trial further demonstrated that axillary radiotherapy achieved comparable regional control to ALND with lower morbidity [9]. The SENOMAC trial, enrolling 2,540 patients with clinically node-negative T1-T3 breast cancer and one or two SLN macrometastases (including mastectomy patients), demonstrated non-inferiority of SLNB alone versus cALND for five-year recurrence-free survival (89.7% vs. 88.7%; hazard ratio (HR) = 0.89), substantially broadening the evidence base for de-escalation [10]. The primary results of the INSEMA trial further showed that omission of SLNB itself did not affect five-year invasive disease-free survival (HR = 0.98) in clinically node-negative patients with T1-T2 tumors, while significantly reducing lymphedema rates [11]. However, the INSEMA secondary analysis specifically in patients with SLN macrometastases showed a non-significant trend favoring cALND (five-year invasive disease-free survival (iDFS) = 86.6% vs. 93.8%; HR = 1.69; p = 0.058), indicating that this question remains incompletely resolved in this subgroup [12]. Reflecting the overall body of evidence, current National Comprehensive Cancer Network (NCCN) and American Society of Clinical Oncology (ASCO) guidelines support omission of cALND in selected patients with T1-T2 tumors, one or two positive SLNs, and planned systemic therapy and radiotherapy [2,13].

Despite this trend toward de-escalation, cALND remains indicated in specific clinical situations, including patients with higher nodal burden or those not meeting established trial eligibility criteria. Understanding the actual rate and extent of additional non-sentinel axillary nodal involvement in patients undergoing cALND remains clinically relevant, as it informs both patient counseling and surgical planning [14,15].

This study aimed to describe the frequency of additional non-sentinel axillary metastases following cALND in patients with a positive SLN at a single center, and to explore clinicopathological associations with additional nodal burden. This study was designed as a descriptive and exploratory retrospective analysis and does not aim to establish predictive models or make causal inferences.

## Materials and methods

Study design and population

This was a retrospective, single-center, observational study conducted at a large public hospital in Attica, Greece, from January 2018 to December 2019. The study population comprised female patients with histologically confirmed breast cancer who underwent surgical treatment, including SLNB, during the study period and were found to have a positive SLN either intraoperatively (frozen section) or on final histopathological examination.

Patient and tumor characteristics

Data were retrieved from the institutional oncology registry. The following variables were recorded: age, type of breast surgery performed (BCS/lumpectomy or mastectomy), laterality of surgery, number of SLNs removed and number of positives, number of axillary lymph nodes removed during cALND and number of positives, pathological TNM tumor stage (T stage), nodal stage (N stage), histopathological subtype, tumor grade (Scarff-Bloom-Richardson grading system), hormone receptor (HR) status (estrogen receptor (ER) and progesterone receptor (PR)), human epidermal growth factor receptor 2 (HER2) status, and Ki-67 proliferation index. Molecular subtypes were defined as luminal (HR+/HER2-), HR+/HER2+, HER2-enriched (HR-/HER2+), and triple negative (HR-/HER2-).

Importantly, data on lymphovascular invasion (LVI) were not available in the institutional registry, and this represents a recognized limitation of the present study. No patient in this cohort received neoadjuvant systemic therapy before surgery; therefore, neoadjuvant treatment status is not a confounding variable in this dataset. These limitations are discussed further in the Limitations section.

SLN identification was performed using either a radiotracer (radioisotope technique), blue dye (methylene blue), or both (dual technique) according to institutional practice and surgeon preference. The dual technique, which achieves sentinel node identification rates exceeding 95% and lower false-negative rates, is considered the gold standard and was employed when feasible [14-16].

Primary and secondary outcomes

The primary outcome was the presence of metastatic involvement in non-sentinel axillary lymph nodes following cALND in patients with a positive SLN. Secondary outcomes included the total number of lymph nodes removed, the number of positive lymph nodes, and associations between clinicopathological characteristics, including surgery type, tumor stage, grade, HR status, HER2 status, and molecular subtype, and axillary lymph node involvement.

Sample size

Because this was a retrospective study, no a priori sample size calculation was performed. All consecutive eligible patients treated during the study period were included. The minimum required sample size for estimating a proportion with 95% confidence, 50% expected proportion, and 10% margin of error was n = 96.04 (formula: n = Z² × p × (1-p) / d²). The final sample of 102 patients was therefore considered adequate for the primary descriptive objective [16].

Statistical analysis

Descriptive statistics were performed for all variables. Continuous variables are presented as mean ± standard deviation (SD), and categorical variables as frequencies and percentages. Normality was assessed using the Kolmogorov-Smirnov test. Independent samples t-test or Mann-Whitney U test were used for continuous variable comparisons; chi-square or Fisher’s exact test for categorical variables; and one-way analysis of variance or Kruskal-Wallis test for multi-group comparisons. A p-value <0.05 was considered statistically significant. All analyses were conducted using SPSS Statistics version 22.0 (IBM Corp., Armonk, NY, USA). Given the exploratory nature of this study and the limited sample size, no multivariable analysis was performed; findings should be interpreted as hypothesis-generating.

Ethical considerations

The study was conducted in accordance with the Declaration of Helsinki and applicable data protection regulations (GDPR). All patient data were anonymized before analysis and used exclusively for research purposes.

## Results

Patient and tumor characteristics

A total of 102 female patients with histologically confirmed breast cancer and a positive SLN were included. The mean age was 57.2 ± 13.2 years (range = 28-85). Regarding the initial surgical procedure, 41 (40.2%) patients underwent right SLNB, 40 (39.2%) patients underwent left SLNB, 14 (13.7%) patients underwent left axillary SLN removal, and seven (6.9%) patients underwent right axillary SLN removal.

During the initial surgical procedure, a mean of 3.01 ± 2.4 SLNs were removed, of which 1.7 ± 1.3 were positive. Additionally, a mean of 11.4 ± 5.2 axillary lymph nodes were removed during cALND, of which 2.6 ± 3.7 were found to be positive. Overall, a total of 14.3 ± 5.4 lymph nodes were removed per patient, with a mean of 4.4 ± 4.1 positive lymph nodes (Table 1).

**Table 1:** Patient and lymph node characteristics. Continuous variables are presented as mean ± standard deviation (SD), with minimum and maximum values also reported. Categorical variables are presented as numbers (N) and percentages (%).

Variable	Minimum	Maximum	Mean ± SD
Age (years)	28	85	57.2 ± 13.2
Sentinel lymph nodes removed	1	18	3.01 ± 2.4
Positive sentinel lymph nodes	1	10	1.7 ± 1.3
Axillary lymph nodes removed	2	27	11.4 ± 5.2
Positive axillary lymph nodes	0	19	2.6 ± 3.7
Total lymph nodes removed	4	30	14.3 ± 5.4
Total positive lymph nodes	1	20	4.4 ± 4.1

Regarding additional patient and tumor characteristics (Table 2), 79.4% of patients (n = 81) underwent BCS/lumpectomy and 20.6% (n = 21) underwent mastectomy. Tumor staging revealed T1 disease in 32.4% (n = 33), T2 in 46.1% (n = 47), and T3 in 5.9% (n = 6); staging data were unavailable for 15.7% (n = 16). The predominant molecular subtype was luminal (HR+/HER2-) in 75.5% (n = 77), followed by HER2+ (HR+) in 8.8% (n = 9), triple negative in 8.8% (n = 9), and HER2-enriched (HR-/HER2+) in 6.9% (n = 7). Tumor grade was 2 in 52.9% (n = 53), grade 3 in 42.2% (n = 43), and grade 1 in 2.0% (n = 2); grade data were missing in 3.9% (n = 4). No patient received neoadjuvant chemotherapy before surgery. LVI data were not recorded in the institutional registry.

**Table 2:** Additional patient and tumor characteristics (N = 102). BCS = breast-conserving surgery; HR = hormone receptor; HER2 = human epidermal growth factor receptor 2; LVI = lymphovascular invasion

Variable	N (%)
Surgery type: BCS/lumpectomy	81 (79.4%)
Surgery type: mastectomy	21 (20.6%)
Tumor stage T1	33 (32.4%)
Tumor stage T2	47 (46.1%)
Tumor stage T3	6 (5.9%)
Tumor stage unknown	16 (15.7%)
Grade 1	2 (2.0%)
Grade 2	53 (52.9%)
Grade 3	43 (42.2%)
Grade unknown	4 (3.9%)
Molecular subtype: luminal (HR+/HER2-)	77 (75.5%)
Molecular subtype: HER2+ (HR+)	9 (8.8%)
Molecular subtype: triple negative	9 (8.8%)
Molecular subtype: HER2-enriched (HR-/HER2+)	7 (6.9%)
Neoadjuvant chemotherapy	0 (0%)
LVI data available	0 (0%) — not recorded in the registry

Axillary lymph node involvement following cALND

Additional non-sentinel axillary lymph node metastases were identified in 60 out of 102 (58.8%) patients, while 42 (41.2%) patients had no further nodal disease beyond the SLN. It is important to emphasize that the majority of patients (58.8%) still harbored additional metastatic axillary nodes, underscoring the continued clinical relevance of ALND in a substantial proportion of SLN-positive patients.

Patients with positive axillary lymph nodes were on average slightly older (58.5 ± 12.8 vs. 55.5 ± 13.6 years; p = 0.267). No statistically significant associations were identified between ALND positivity and surgery type (BCS vs. mastectomy: 72% vs. 88% in ALND+ vs. ALND- groups; p = 0.096), tumor stage, grade, HR status, or molecular subtype (Table 3). The absence of significant associations likely reflects the limited statistical power of this exploratory study rather than a true absence of association, and these findings should be interpreted accordingly.

**Table 3:** Comparison based on axillary lymph node status. Continuous variables are presented as mean ± standard deviation (SD), while categorical variables are presented as numbers (N) and percentages (%). Comparisons between groups were performed using the independent samples t-test for continuous variables and the chi-square test or Fisher’s exact test for categorical variables, as appropriate. Statistical significance was set at p < 0.05. NS = not statistically significant; ALND = axillary lymph node dissection; DCIS = ductal carcinoma in situ; BCS = breast-conserving surgery; HR = hormone receptor; SLN = sentinel lymph node

Variable	ALND positive (n = 60)	ALND negative (n = 42)	Statistical test	P-value
Age	58.5 ± 12.8	55.5 ± 13.6	t = −0.079	0.267
Invasive carcinoma (%)	100%	95.2%	X² = 1.069	0.089
Ductal carcinoma (%)	90%	88.1%	X² = 0.010	0.766
DCIS (%)	20%	19%	X² = 0.263	0.906
Lobular carcinoma (%)	6.7%	7.1%	X² = 1.200	0.926
BCS (%)	43/60 (72%)	38/42 (88%)	X²	0.096
Mastectomy (%)	17/60 (28%)	5/42 (12%)	X²	0.096
Tumor stage T1 (%)	16/60 (27%)	17/42 (40%)	X²	NS
Tumor stage T2 (%)	29/60 (48%)	18/42 (43%)	X²	NS
Tumor stage T3 (%)	5/60 (8%)	1/42 (2%)	X²	NS
Grade 3 (%)	27/60 (45%)	16/42 (38%)	X²	NS
HR+ (%)	48/60 (80%)	38/42 (91%)	X²	NS
Triple negative (%)	7/60 (12%)	2/42 (5%)	X²	NS
Positive SLNs, mean ± SD	1.93 ± 1.51	1.51 ± 0.88	U	0.181

Sentinel node as the sole metastatic site

In patients where the SLN was the only metastatic node, no positive axillary lymph nodes were identified following ALND. These patients had a significantly lower number of total positive lymph nodes (1.5 ± 0.9 vs. 6.4 ± 4.2; p < 0.001) and no additional axillary involvement (0 vs. 4.5 ± 3.9; p < 0.001). Furthermore, these patients had a significantly higher number of SLNs removed (3.6 ± 2.8 vs. 2.6 ± 1.8; p = 0.034) and a lower number of axillary lymph nodes removed (9.7 ± 4.5 vs. 12.5 ± 5.3; p = 0.006). Although not statistically significant, patients in whom the sentinel node was the only involved node showed lower rates of invasive carcinoma and ductal carcinoma (Table 4).

**Table 4:** Sentinel node as the sole metastatic node. Continuous variables are presented as mean ± standard deviation (SD). Comparisons between groups were performed using the independent samples t-test. Statistical significance was set at p < 0.05. SLN = sentinel lymph node; ALN = axillary lymph node

Variable	SLN-only	Additional nodes	Statistical test	P-value
SLNs removed	3.6 ± 2.8	2.6 ± 1.8	−1.615	0.034
ALNs removed	9.7 ± 4.5	12.5 ± 5.3	−8.987	0.006
Positive ALNs	0	4.5 ± 3.9	−8.731	0.001
Total positive nodes	1.5 ± 0.9	6.4 ± 4.2	−1.738	0.001

Histological subtype and nodal burden

Patients with invasive carcinoma had a significantly higher number of positive axillary lymph nodes (2.7 ± 3.7 vs. 0; p = 0.001) and a higher total number of positive lymph nodes (4.4 ± 4.1 vs. 1.5 ± 0.7; p = 0.024), despite having fewer total lymph nodes removed (14.3 ± 5.5 vs. 16.5 ± 0.7; p = 0.031). Patients with ductal carcinoma had a higher number of positive SLNs compared to non-ductal carcinoma cases (1.8 ± 1.3 vs. 1.2 ± 0.4; p = 0.001). No statistically significant associations were identified for other histological subtypes, likely due to small subgroup sizes.

## Discussion

The present study investigated axillary lymph node findings following cALND in a real-world, single-center cohort of 102 patients with positive SLNs. Two findings of equal clinical importance emerge: first, 58.8% of patients had additional non-sentinel axillary metastases after ALND, demonstrating that residual nodal disease is common and clinically significant; and second, 41.2% of patients had no further axillary involvement beyond the SLN, consistent with existing literature and supporting the concept that ALND may be avoidable in selected patients [17,18].

It is important that both findings be considered in context. The observation that 41.2% of patients had no further nodal disease does not in itself demonstrate that ALND was “unnecessary” for those individuals, as this study does not evaluate oncologic outcomes such as recurrence, disease-free survival, or locoregional control. The absence of outcome data is a key limitation, and conclusions regarding the safety of ALND omission must be drawn from prospective randomized trials rather than from retrospective descriptive data [5-8].

The paradigm shift in axillary management has been driven by landmark randomized trials. ACOSOG Z0011 demonstrated that omission of ALND in patients with one or two positive SLNs undergoing BCS with whole-breast radiotherapy did not compromise overall survival or locoregional control [5,6]. The IBCSG 23-01 trial established the safety of omitting ALND in patients with SLN micrometastases [7,8]. The AMAROS trial showed that axillary radiotherapy achieves comparable regional control to ALND with lower morbidity, particularly lymphedema [9]. The SENOMAC trial, the largest trial to date in this setting, enrolled 2,540 patients with T1-T3 clinically node-negative breast cancer and one or two SLN macrometastases, including mastectomy patients. It demonstrated non-inferiority of SLNB alone versus cALND for five-year recurrence-free survival (89.7% vs. 88.7%; HR = 0.89; p < 0.001 for non-inferiority), providing strong evidence supporting de-escalation in this broader population [10]. The primary results of the INSEMA trial, published in the New England Journal of Medicine in 2025, further demonstrated that complete omission of SLNB was non-inferior to SLNB for five-year iDFS (HR = 0.98; 95% confidence interval (CI) = 0.73-1.14) in patients with clinically node-negative T1-T2 breast cancer, while significantly reducing lymphedema rates [11]. Notably, however, the INSEMA secondary analysis in patients with SLN macrometastases showed a non-significant trend favoring cALND (five-year iDFS = 86.6% vs. 93.8%; HR = 1.69; p = 0.058), with non-inferiority of SLNB alone not formally demonstrated in this subgroup [12]. Current NCCN and ASCO guidelines, reflecting the overall evidence base, principally from Z0011, AMAROS, and SENOMAC, support the omission of cALND in carefully selected patients with T1-T2 tumors, one to two positive SLNs, and planned adjuvant radiotherapy and systemic therapy [2,13].

Our cohort predates the broad implementation of these guidelines (2018-2019) and consists exclusively of patients who underwent cALND, introducing a selection bias that limits direct applicability to current practice, where many comparable patients would no longer routinely undergo ALND. This is an important limitation: the conclusions of this study apply specifically to the subset of patients in whom ALND is being performed, not to the broader population of SLN-positive patients managed conservatively in contemporary practice.

Within this cohort, we confirmed that the presence of invasive carcinoma is associated with significantly higher axillary nodal burden, consistent with published literature on tumor biology and lymphatic metastasis [19]. A numerically higher rate of additional nodal involvement was observed in triple-negative patients (12% vs. 5%), though this did not reach statistical significance, likely due to the small number of cases in this subgroup. Similarly, a trend toward higher ALND positivity in patients who underwent mastectomy (compared to BCS) was observed, though not statistically significant, and may reflect disease severity at the time of surgical decision-making.

Reliable predictors of non-SLN involvement remain an active area of investigation. Published nomograms and predictive models, including the MSKCC nomogram and others, incorporate variables such as tumor size, LVI, and number of positive SLNs, but none have been universally validated or integrated into routine practice [15]. In our study, the absence of LVI data and multivariable analysis preclude any contribution to predictive modeling, and this is acknowledged as a significant limitation.

Limitations

This study has several limitations that require emphasis. First, this is a retrospective, single-center study, which limits generalizability. Second, the cohort consists exclusively of patients who underwent cALND, introducing selection bias and limiting applicability to contemporary practice, where many SLN-positive patients are managed conservatively. Third, key tumor biology variables, specifically LVI, were unavailable; no patient received neoadjuvant therapy, precluding analysis of treatment-related effects. Fourth, multivariable analysis was not performed; associations identified are exploratory only. Fifth, and critically, no oncologic outcome data (recurrence, disease-free survival, locoregional control, or overall survival) are available, meaning the clinical implications of having no additional axillary disease cannot be assessed from these data alone. These limitations should be considered when interpreting the findings.

## Conclusions

In this retrospective, single-center study of 102 patients with positive SLNs, 58.8% had additional non-sentinel axillary metastases following cALND, a clinically important finding demonstrating that residual nodal disease remains common. Conversely, 41.2% had no further axillary involvement, consistent with current evidence supporting de-escalation of axillary surgery in carefully selected patients. The presence of invasive carcinoma was associated with a higher nodal burden. No statistically significant associations between ALND positivity and surgery type, tumor stage, tumor grade, HR status, or molecular subtype were identified, likely reflecting the limited statistical power of this exploratory study. These findings should not be interpreted as demonstrating that ALND was unnecessary in the 41.2% of patients without additional nodal disease, as no oncologic outcome data are available. Rather, they support the individualized approach to axillary surgery endorsed by current guidelines and landmark trials (Z0011, IBCSG 23-01, AMAROS, SENOMAC), in which patient-specific factors, including tumor biology, planned adjuvant therapy, and nodal burden, guide surgical decision-making. Future prospective studies incorporating tumor biology, LVI, and long-term oncologic outcomes are needed to refine patient selection for omission of cALND.
